# A modified method for enzymatic isolation of and subsequent wax extraction from *Arabidopsis thaliana* leaf cuticle

**DOI:** 10.1186/s13007-020-00673-7

**Published:** 2020-09-21

**Authors:** Martina Vráblová, Daniel Vrábl, Barbora Sokolová, Dominika Marková, Marie Hronková

**Affiliations:** 1 VSB-Technical University of Ostrava, Institute of Environmental Technology, 17. listopadu 15, 708 00 Ostrava, Czech Republic; 2 University of Ostrava, Faculty of Science, Chittussiho 10, 710 00 Ostrava, Czech Republic; 3 VSB-Technical University of Ostrava, Faculty of Materials Science and Technology, 17. listopadu 15, 708 00 Ostrava, Czech Republic; 4 Biology Centre of Czech Academy of Sciences, Institute of Plant Molecular Biology, Branisovska 31, 370 05 Ceske Budejovice, Czech Republic; 5 University of South Bohemia, Faculty of Science, Branisovska 1760, 370 05 Ceske Budejovice, Czech Republic

**Keywords:** *Arabidopsis thaliana*, Leaf cuticle, Enzymatic isolation, Fatty acids, Wax

## Abstract

**Background:**

The plant cuticle represents one of the major adaptations of vascular plants to terrestrial life. Cuticular permeability and chemical composition differ among species. *Arabidopsis thaliana* is a widely used model for biochemical and molecular genetic studies in plants. However, attempts to isolate the intact cuticle from fresh leaves of Arabidopsis have failed so far. The goal of this study was to optimise an enzymatic method for cuticle isolation of species with a thin cuticle and to test it on several *A. thaliana* wild types and mutants.

**Results:**

We developed a method for isolation of thin cuticles that allows reducing the isolation time, the separation of abaxial and adaxial cuticles, and avoids formation of wrinkles. Optical microscopy was used for studying cuticle intactness and scanning electron microscopy for visualisation of external and internal cuticle structures after isolation. Wax extracts were analysed by GC–MS. Isolation of intact cuticle was successful for all tested plants. The wax compositions (very-long-chained fatty acids, alcohols and alkanes) of intact leaves and isolated cuticles of wild type Col-0 were compared.

**Conclusions:**

We conclude that the optimised enzymatic method is suitable for the isolation of *A. thaliana* adaxial and abaxial cuticles. The isolated cuticles are suitable for microscopic observation. Analysis of wax composition revealed some discrepancies between isolated cuticles and intact leaves with a higher yield of wax in isolated cuticles.

## Background

The cuticular membrane covering the epidermis is an essential structure, protecting the aerial surface of living cells in all land plants. It is the lipophilic product of the epidermal cells of leaves, primary stems and fruits. Cuticles also occur internally in the lining of substomatal cavities. The primary function of the cuticle is to prevent uncontrolled water loss and regulation of gas exchange. Besides that, it works as a natural UV filter and as an infection barrier for viruses, bacteria and fungi. Moreover, the cuticle plays an important role as a border preventing organ fusion during organogenesis [23, 25, 29].

Cuticles consist of an insoluble matrix and wax soluble in organic solvents, which form layers above the cell wall or, in a new concept, are understood as a lipidised, chemically and structurally heterogeneous region of the epidermal cell wall [9]. The matrix is formed by cutin, possibly cutan and/or polysaccharides [26, 35]. The wax comprises linear very-long-chain compounds: acids, primary and secondary alcohols, alkanes, aldehydes, ketones, esters, and possibly cyclic compounds [16]. Two forms of wax can be distinguished [13]: epicuticular wax is the outermost layer and can be physically stripped off [7, 28], whereas intracuticular wax is embedded in the cutin matrix and can only be extracted with organic solvents; its chemical composition can differ from epicuticular wax [7]. Knowledge about extra- and intracuticular wax composition is essential for understanding the role of the cuticle as transpiration barrier [17, 28].

The cuticular membrane is being studied from different perspectives. Water and CO_2_ permeability is of interest to plant physiologists and ecologists studying diffusional limitations of photosynthesis and water use efficiency [3, 6, 30]. Knowledge of cuticle permeability for mineral nutrients and pesticides is essential for agrochemists in order to apply the proper concentrations of active ingredients and solvents [20, 31]. Phytopathologists are focused on the physical and chemical properties of cuticles that affect the phyllosphere—the leaf surface environment, where viruses, bacteria, fungi, yeasts and insects interact with the plant [2]. Molecular biologists are interested in the identification of promoters, genes and signals involved in cuticle biosynthesis and deposition [22, 37]. Moreover, the chemical composition and structure of cuticular membranes can provide inspiration to polymer chemists to create cutin-based materials [11].

Enzymatic isolation is a commonly used method for peeling off the cuticular membrane. A solution of pectinase and cellulase is used. Pectinase digests the pectin layer interposed between the cuticle and the cellulose cell wall of the epidermis. The function of cellulase is similar in that it digests the cellulose in cell walls but the benefit of including it has never been clearly demonstrated [33]. An effect of the isolation process on cuticle properties, especially in relation to its chemical composition, has been demonstrated [32]. Therefore, extraction with organic solvents directly from fresh leaves is often used for wax chemical analysis [24, 28]. However, enzymatic isolation is currently the only tool we have to obtain intact cuticles appropriate for permeability measurements [7, 9, 18, 38].

A major limitation of cuticle research is the inability to isolate cuticular membranes from various plant species. Many cuticles are too delicate to be isolated, such as the cuticle from non-vascular plants and some angiosperms [13]. Furthermore, some species are recalcitrant to enzymatic isolation, probably due to the higher content of phenolic compounds in the epidermal cell wall. In particular, the inability to properly isolate the cuticle of *Arabidopsis thaliana*, the traditional biochemical and molecular genetic model plant, significantly limits studies of cuticle transport properties, development and signalling [34]. In these cases, cuticle research is largely restricted to chemical analysis of cuticular wax obtained from intact leaves, which can be extracted with non-polar solvents such as chloroform, but where there is always a risk that some products obtained by washing the surface with solvents may originate from inner parts of the leaf, especially when the extraction is done from a stomatous leaf surface [4].

Solvent extraction of cuticle wax from *A. thaliana* leaves is usually applied when the chemical composition of wax is studied. Due to the thickness of the Arabidopsis cuticle, which ranges from 15 to 29 nm [10], many of the attempts to isolate intact Arabidopsis cuticles have failed. To the best of our knowledge, no protocol for isolation of cuticular membranes from fresh leaves of *A. thaliana* is available, though Franke et al. [10] applied the enzymatic method to isolate intact cuticles from dried Arabidopsis leaf discs.

In this work, we adapted the original enzymatic method, which enables isolation of the cuticular membrane from plant species with very delicate cuticles, such as *A. thaliana*. With this adaptation, intact abaxial and adaxial cuticular membranes can be isolated and used for chemical analysis without ambiguities concerning the origin of extracted compounds.

## Results

**Fig. 1:**
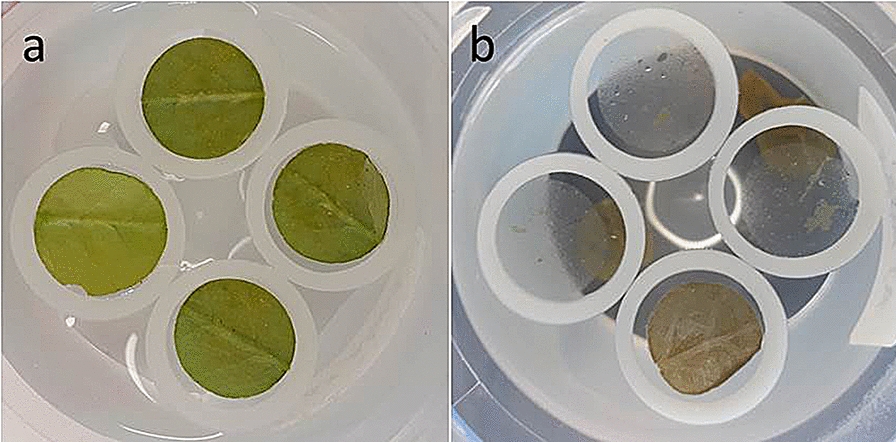
Design of an optimised method for enzymatic isolation of Arabidopsis leaf cuticle. Fresh leaf discs floating on solution surface (**a**) and digested leaves with isolated cuticles after approximately 1 week of isolation (**b**) are shown. Silicone annuli were used to keep cuticle discs floating on the water surface, whereas digested tissue sank down

**Fig. 2:**
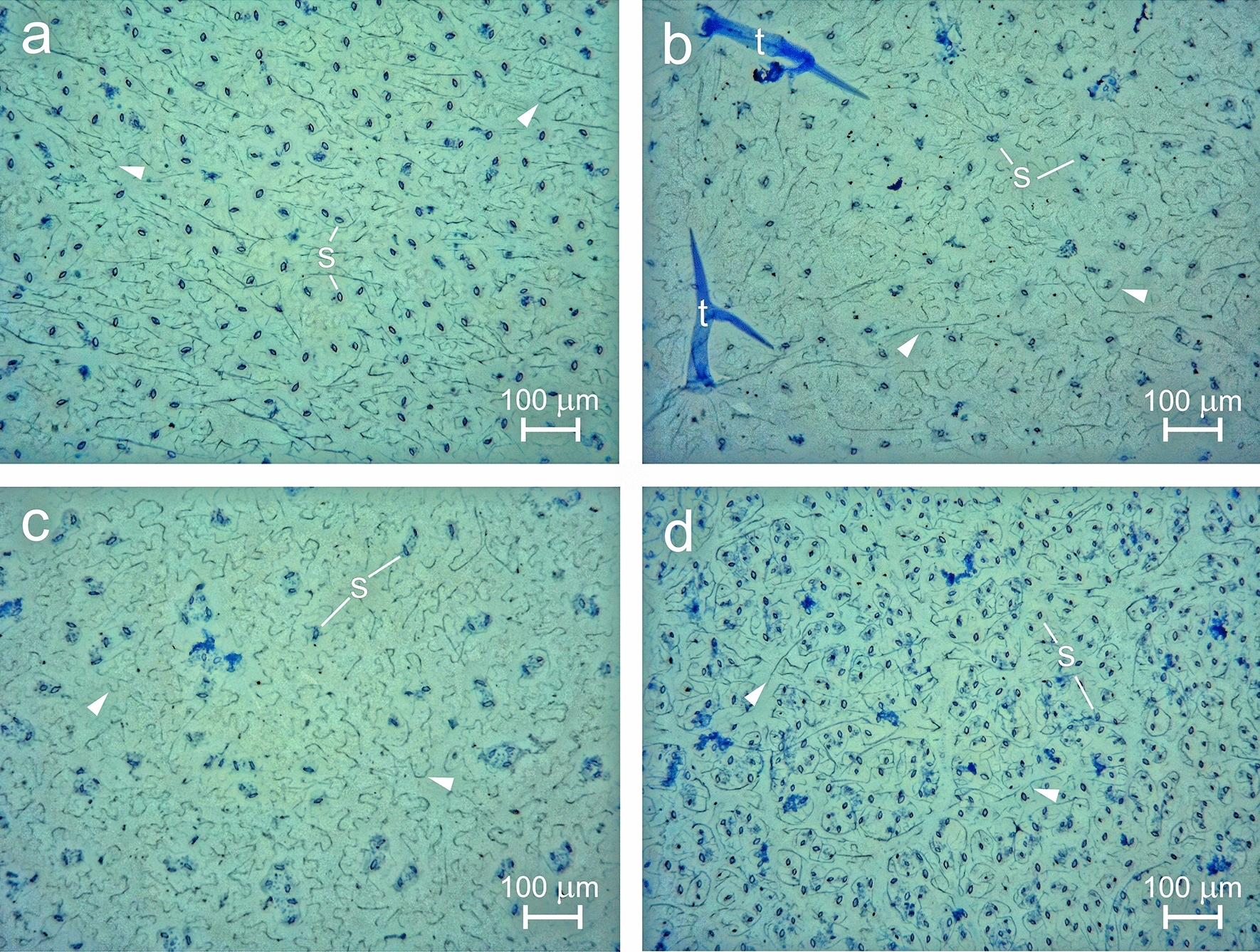
Optical microscopy of isolated abaxial cuticles from Col-0 (**a**), L*er* (**b**), *tmm1*-*1* (**c**) and *epf1,2* (**d**). Cuticles were stained with aqueous Nile Blue A. Outlines of stomatal guard cells and pores (s), trichome envelopes (t) and borders of formerly present epidermal cells (arrowheads) are visible in dark blue

Adaxial and abaxial cuticles of *A. thaliana* were successfully isolated enzymatically from leaves of wild types Col-0 and L*er*, and several mutants (*epf1,2, StRNAi, StOx* and *tmm1*-*1*). Green leaf tissue dropped to the bottom of the containers during the isolation process, whereas clear cuticles stayed floating on the surface of the enzymatic solution (Fig. 1). Optical microscopy confirmed the integrity of all cuticles. Examples of Nile blue-stained abaxial cuticles of Col, L*er*, *tmm1*-*1* and *epf1,2* are shown in Fig. 2a–d. The blue stain was clearly visible along the borders of epidermal pavement cells, and over stomata or stomatal clusters and trichomes. No cracks or holes were found.

**Fig. 3:**
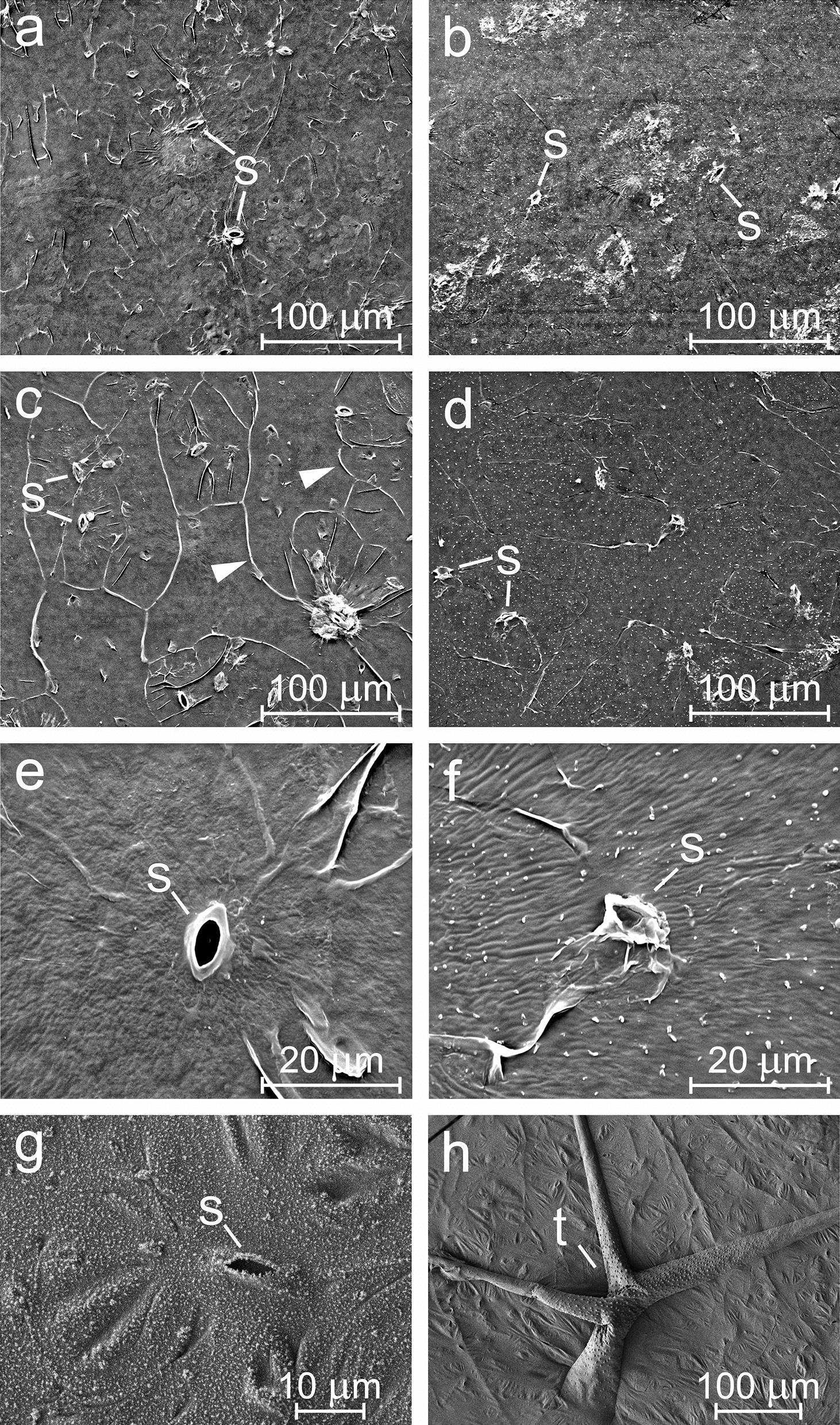
Scanning electron microscopy (**a**–**f**) and cryomicroscopy (**g**–**h**) of isolated cuticles of *Arabidopsis thaliana* Col-0 wild type. Adaxial external (**a**) and internal (**b**), abaxial external (**c**) and internal (**d**) sides of cuticles observed in secondary electron mode are shown. Details of the relief of abaxial cuticle and a stoma seen at higher magnification from the external (**e**) and internal (**f**) side, respectively, are included to show that a cuticle was present in substomatal cavities (**f**). Adaxial cuticles observed by cryomicroscopy are shown (**g**, **h**). Outlines of stomatal guard cells and pores (s), trichome envelopes (t), and borders of formerly present epidermal cells (arrowheads) are visible

Special attention was paid to the cuticle of Col-0, which was thereafter used for wax analysis. Both air-facing (external) and mesophyll-facing (internal) surfaces of the abaxial and adaxial cuticles were studied by scanning electron microscopy (SEM) (Fig. 3) and cryoSEM. The external cuticle surface (facing the atmosphere) was almost smooth with no discernible epicuticular wax structures. At the inner side (facing the mesophyll), the cuticular lining of substomatal cavities was visible. Attempts to obtain images of cuticular cross sections were made to confirm its thickness. Artificially created cross sections were hard to realise due to low thickness and high adhesion of the cuticle. Nevertheless, at naturally occurring incisions of the cuticle we were able to measure cuticle thickness, which was *c*. 70–80 nm. This is in agreement with Kosma et al. [21] who obtained values of 66–82 nm in cross sections of fixed native leaves observed under TEM microscopy.

Qualitative analysis of the elemental composition of cuticles was performed in SEM. Only C and O were found across most of the cuticle surface, which corresponds with the composition of wax (long-chain hydrocarbons, acids, aldehydes, alcohols or esters) [15]. When we focused on small areas with some remaining tissue, which was occasionally found on the inner side of some samples, we found C, O, N, P and S, which corresponds with the chemical composition of compounds in cell walls (e.g. proteins) [1].

**Fig. 4:**
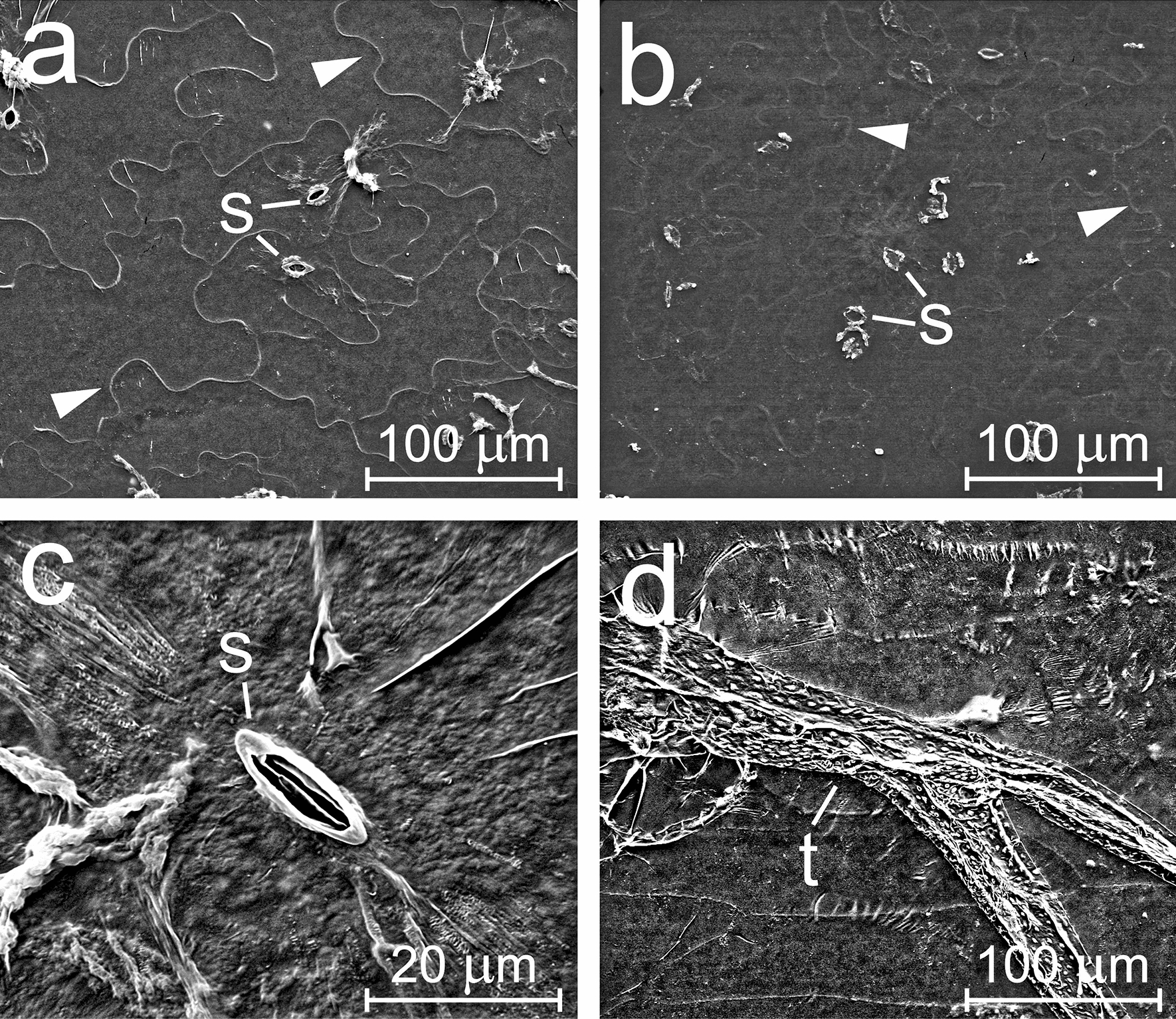
Scanning electron microscopy of isolated cuticles of *Arabidopsis thaliana* Col-0 wild type, treated (washed) on the external side with chloroform before enzymatic isolation. External (**a**) and internal (**b**) side of abaxial cuticle, and details of adaxial cuticle with a stoma (**c**) and of a trichome envelope (**d**) are shown. Outlines of stomatal guard cells and pores (s), trichome envelopes (t) and borders of formerly present epidermal cells (arrowheads) are visible

SEM images were also collected from cuticles isolated after first washing leaf surfaces with CHCl_3_. This allowed us to obtain cuticles without epicuticular wax (Fig. 4). While the external surface of cuticles was exposed to CHCl_3_ directly, the inner surface remained in contact with the rest of the leaf during the CHCl_3_ treatment. Leaf discs were placed into enzymatic solution immediately after CHCl_3_ treatment (with the CHCl_3_-treated side of the cuticle oriented upwards during the isolation). A possible effect of CHCl_3_ on the inner cuticular surface cannot be excluded, however, especially in areas close to stomatal pores. After CHCl_3_ treatment, epidermal cell borders appeared to be more clearly visible and white dots on the inner side of cuticles (see Fig. 3d, f) were eliminated.

**Fig. 5:**
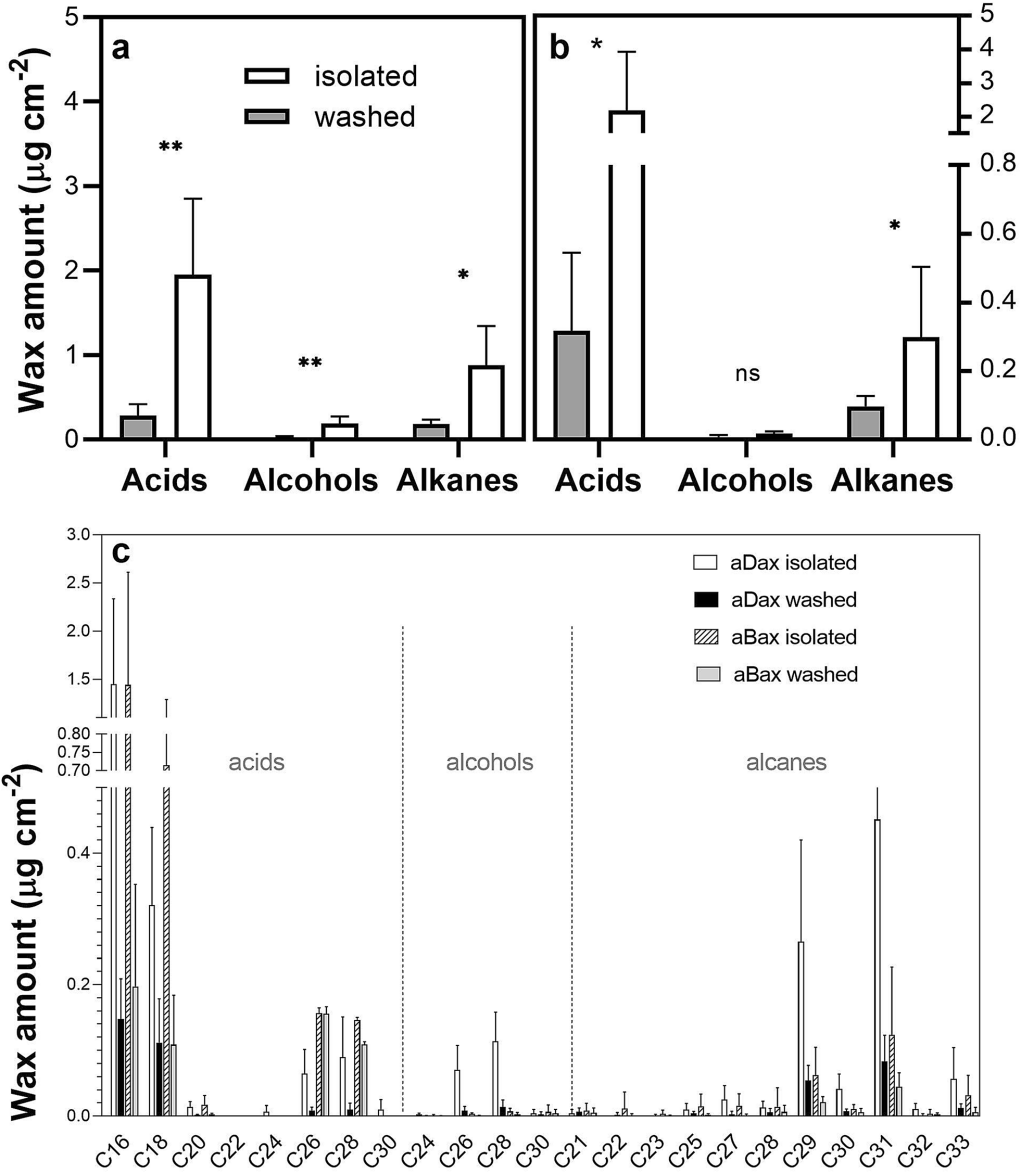
Wax amount per unit area of extracted isolated cuticles and washed leaf surfaces of *A. thaliana* Col-0 wild type. C16–C30 acids, C24–C30 alcohols and C20–C33 alkanes were evaluated separately from adaxial (**a**) and abaxial (**b**) cuticles/leaf surfaces. Values for individual compounds on both leaf sides are also shown (**c**). Mean values from five replicates are shown. On average, wax from 20 leaves from 12 plants was pooled in any one replicate to make up 20 cm^2^ of cuticle area. Bars represent one standard deviation. Symbols: ns p > 0.05; *p ≤ 0.05; **p ≤ 0.01; ***p ≤ 0.001

**Fig. 6:**
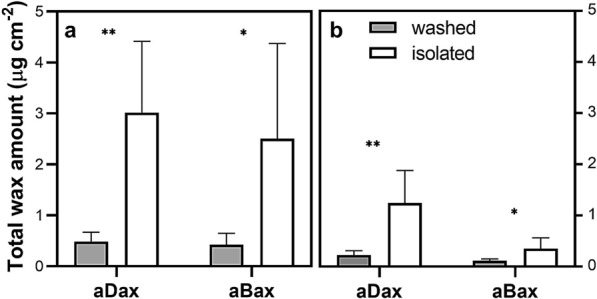
Total wax amount per unit area of isolated and washed adaxial and abaxial cuticles of *A. thaliana*. Total wax amount was calculated as the sum of C16–C30 acids, C24–C30 alcohols and C20–C33 alkanes (**a**). Total wax amount after omission of C16-C18 acids (**b**). Mean values from 5 replicates are shown. On average, wax from 20 leaves from 12 plants of Col-0 ecotype was collected in one replicate to make up 20 cm^2^ of cuticle area. Bars represent one standard deviation. Symbols: ns p > 0.05; *p ≤ 0.05; **p ≤ 0.01; ***p ≤ 0.001

Chemical analysis of wax composition was focused on three functional groups—long-chain fatty acids (C16–C30) and the derived alcohols (C24–C30) and alkanes (C20–C33) (Fig. 5). Among the different functional groups of wax compounds, acids comprised the largest portion and alcohols the smallest (Fig. 5). Within each group, the most abundant compounds were C16 acid, C28 alcohol and C31 alkane, respectively (Fig. 5c). The total amounts of wax extracted from isolated adaxial and abaxial cuticles after 24 h were 3.0 and 2.5 μg cm^−2^, respectively, whereas the amounts obtained by CHCl_3_ extraction from intact leaves (washing for 30 s) were significantly lower (0.49 and 0.42 μg cm^−2^ for adaxial and abaxial cuticles, respectively) (Fig. 6a). We found differences in the amount of total wax extracted from isolated cuticles and intact leaf surfaces even when the C16 and C18 acids (the most abundant wax acids to be found also in leaf tissue [12]) were eliminated from the calculation (Fig. 6b). A moderate correlation between the amounts of individual compounds from isolated and CHCl_3_-washed cuticles was found (k = 5.3, R^2^ = 0.70). Therefore, the isolation process and subsequent wax extraction strongly affected the results for total quantities of wax compounds but had only a weak effect on their relative composition.

Chemical analysis of cuticles pre-treated with CHCl_3_ and subsequently isolated revealed that the amount of wax extracted after isolation was comparable to or higher than that obtained by the leaf washing the leaf with CHCl_3_ (0.009 vs. 0.006 μg cm^−2^ alcohols, 0.078 vs. 0.088 μg cm^−2^ alkanes, 3.169 vs. 0.468 μg cm^−2^ acids, or 0.033 vs. 0.009 μg cm^−2^ acids when C16 and C18 acids were omitted). This shows that the amount of wax extracted from isolated, previously CHCl_3_-washed cuticles was not negligible and could correspond to the presence of intracuticular wax.

## Discussion

Isolation of Arabidopsis cuticle is difficult because it is not only fragile but also much less than 100 nm thick [10, 21]. Franke et al. [10] isolated Arabidopsis cuticles from dried leaf discs that were incubated with cellulase and pectinase, but this method results in wrinkled cuticular membranes. Our previous experience with isolation of thicker cuticles (up to 10 μm, e.g. that of *Ficus elastica* or *Hedera helix*) led us to develop this method, which allows (i) shorter isolation time, (ii) separate isolation of abaxial and adaxial cuticles and (iii) absence of wrinkles in the isolated membrane. The first two of these points were achieved by mechanical disruption of the leaf surface opposite to that carrying the desired cuticle, and the third by keeping the cuticle floating on the water surface. The surface tension of water does not allow the cuticle to crease, and the arrangement with the internal side of the cuticle being hydrated and the external side surrounded by air prevents the contact of wax with the isolation medium. This contributes to good membrane quality even after isolation.

Images obtained by optical and electron microscopy revealed the intactness of the isolated cuticle. The borders of pavement cells and guard cells, lining of stomatal pores and substomatal cavities, and envelopes of trichomes were clearly visible. No epicuticular wax crystals were observed. An amorphous layer rather than crystalline wax structures has also been observed in SEM images of intact leaves of *A. thaliana* [8, 15].

The total amount of cuticular wax was estimated to be 0.91 μg cm^−2^ when a non-polar solvent (chloroform) was used for its extraction from intact leaves. This is in good agreement with previously published results (0.7 μg cm^−2^ [21]; 0.9 μg cm^−2^ [7]; 1.2 μg cm^−2^ [5]). Isolation of the cuticle led to a higher yield of extracted wax. For isolated cuticles, the contact time with CHCl_3_ for wax extraction was long (24 h). On the other hand, contact time with CHCl_3_ during washing of leaf surfaces was limited and it was very difficult to estimate the length of contact time sufficient for complete wax extraction without contamination by compounds originating from the leaf interior. We chose a contact time for washing of 30 s, similar to Kim et al. [19], whereas other authors used a longer (2 × 30 s) [7] or shorter (10 s) [27] time when extracting wax from Arabidopsis leaves. Bourgault et al. [5] tested several solvents and extraction times (0.5, 1, 2 and 5 min) and found no significant differences in the wax amount. On the other hand, Bewick et al. [4] found that a much longer time was needed for extraction of epicuticular hydrocarbons from the leaf surface of *Solanum americanum* (up to 6 h) and *Panicum repens* (up to 1 h). It is obvious that for proper wax extraction, the duration of contact between cuticle and solvent is important, and cuticle isolation allows increased contact time and simplification of extraction steps, which are usually more complicated [14].

However, the isolation process can lead to resorption of lipophilic compounds from the enzymatic solution by the cuticle. It has been shown that the adsorbed amount can be up to 22% of the weight of the cuticular matrix [32]. In this case, the authors tested resorption on a wax-free cuticular matrix. Usually, wax remains on the cuticle during isolation and its presence can reduce the resorption of lipophilic compounds. Other factors that can influence the adsorption process are the ratio of solution volume to membrane area, concentration of enzymes and digestate in solution, etc. Therefore, we maximised the volume of the solution used for extraction and put only a limited number of leaves into one container.

## Conclusions

Successful isolation of the Arabidopsis cuticle provides new opportunities for further research. We believe that the extraction of wax from isolated cuticles refines the determination of the amount and composition of total wax and its different chemical groups. Moreover, other chemical analyses (e.g. estimation of phenols) and research in physicochemical properties (UV absorption, permeability to water or organic compounds) will be possible with intact cuticles of *A. thaliana*. Our new method for cuticle isolation is a first but essential step towards the above-mentioned applications.

## Methods

### Plant material

*Arabidopsis thaliana* (L.) Heynh. wild type Col-0 was chosen for isolation of leaf cuticles. Plants were cultivated in pots with soil in a growth chamber at a photon flux density of 100 μmol m^−2^ s^−1^ in an 8: 16 h light: dark cycle at 20 °C and 60% RH. Fully developed leaves from plants at least 8 weeks old were used for cuticle isolation.

The same isolation process was used for another Arabidopsis wild type—ecotype L*er* (Landsberg *erecta*)—and Arabidopsis mutants *epf1*,*2*, *StOX*, *St RNAi* and *tmm1*-*1*, which have altered stomatal densities (SD) and/or distribution patterns. Compared to Col-0, with SD of 140–200 mm^−2^, *Epf1*,*2* (double mutant in two main negative epidermal factors/EPFs) has twice higher SD. *StRNAi* (line silenced in *STOMAGEN*-*EPFL9*) has half the SD and StOx (*STOMAGEN* overexpressed line) has increased SD. *Tmm1*-*1* has loss-of-function mutations in the *TMM* gene [36] with changed stomatal pattern, low SD on the adaxial side, and high SD with frequent stomatal clusters (groups of 2-4 stomata) on the abaxial side. All plants were cultivated under the same conditions as Col-0.

### Cuticle isolation

The solution for isolation of cuticular membranes was prepared as described by Schönherr and Riederer [32] with some modifications. The chemical composition of the solution—buffer and active enzymes—was not changed: 645 mg of citric acid monohydrate and 3 ml of 1 M sodium azide were dissolved in 300 ml of distilled water. The acidity of the solution was adjusted by adding citric acid or KOH to pH = 3. Then 6 ml 2% cellulase (Sigma-Aldrich—Cellulase Onozuka R-10) and 6 ml 2% pectinase (Fruikozyme Combi, Harapes, Czech Republic, containing pectin lyase, polygalacturonase, and pectin esterase) was mixed with the solution. The molarity of the resulting solution was changed compared to the original method; in order to isolate cuticular membranes from Arabidopsis and other plant species with thin cuticles the enzymatic solution prepared as mentioned above should be diluted ten times with distilled water, otherwise the tissue will decompose too quickly and the cuticle will be damaged.

Discs (0.8–2.0 cm in diameter) or rectangles (maximum 1.5 × 2.5 cm) were cut from leaf blades. Mechanical destruction of the leaf cuticle that was not going to be isolated was necessary to allow easier penetration of the enzymatic solution into the leaf interior. Fine sandpaper was used for subtle mechanical abrasion of the unwanted side of the leaf by hand. We do not recommend vacuum infiltration, to avoid damage to the cuticle. Leaf discs were gently placed on the surface of the enzymatic solution in glass or plastic containers (volume 100 ml, diameter 55 mm, for max. 4 leaf discs of 12 mm diameter). It was necessary that leaf discs floated on the solution surface during the whole isolation process. If the disc sank the tissue was broken and the cuticle twisted and torn. A silicone underlay with round holes or silicone annuli helped to keep the cuticle on the surface. The isolation containers were stored at laboratory temperature (23 °C) in darkness.

The isolation time depended on the degree of leaf surface disintegration on the solution-facing side. On average, the abraded leaf side tissue sank to the bottom after 1–2 weeks while the cuticular membrane remained floating on the surface of the enzymatic solution. Once the cuticle was detached from the epidermis and any remaining mesophyll tissue, the enzymatic solution was replaced with distilled water. The isolated cuticular membrane could be stained for optical microscopy with the water soluble stain Nile Blue A (Nile blue sulfate from Sigma-Aldrich) at a final concentration of 0.06 mg/ml. To avoid unnecessary manipulation of the fragile cuticles, the stain was added directly to the isolation container.

### Optical microscopy

A brightfield optical microscope (VisiScope, VWR Collection) was used to ascertain whether cuticular membranes were intact and clean (any remains of the associated cell walls and plasma membrane contaminants were undesirable). A disadvantage of the bright field technique is the low contrast of the image. To increase the cuticle contrast, it was stained with Nile Blue A after isolation. However, for other purposes (chemical composition or diffusion measurements), staining is undesirable. Samples were moved onto the microscope slide directly from the water surface and were allowed to dry in air under laboratory conditions. Abaxial and adaxial cuticles, clear or stained blue, were prepared for optical microscopy.

### Scanning electron microscopy

For microscopic investigations of the cuticle surface, a scanning electron microscope (SEM: FEI Quanta 450) with field emission gun (FEG) was used. Micrographs were obtained using secondary electrons (SE) mode with an acceleration voltage of 20 keV. Cuticle samples were moved directly from the water surface onto a metal holder that was covered with sticky carbon tape. The orientation of cuticles on the water surface was always with the external side on top (originally facing the atmosphere and surrounded by air during the isolation process) and the internal side facing downwards (originally facing the leaf tissue and surrounded by enzymatic solution during the isolation process). For SEM analysis, first the internal and then the external side of cuticles was placed on holders by touching the floating cuticles with the holder from the required direction. After contact with the holder, the cuticle adhered to the sticky carbon tape and was removed from the solution. Therefore, the side of the cuticle that did not touch the holder was visible. Then the sample was air-dried under laboratory conditions. Finally, dry samples were gold coated in order to ensure adequate electron conductivity and placed into the SEM chamber. Both abaxial and adaxial cuticles were analysed.

### Scanning electron cryomicroscopy

A high-resolution scanning electron microscope was used, which was equipped with a cryo-attachment (cryoSEM) that represents the cutting edge technique for examination of wax-containing specimens as close as possible to their native state.

Small pieces (discs) of enzymatically isolated cuticle from Arabidopsis leaves, floating on the surface of water, were placed on the silicon wafer (5 × 7 mm) and immediately and rapidly frozen in liquid nitrogen. The specimen was transferred under vacuum into the cryo-attachment (CryoALTO 2500, Gatan, UK), where vacuum sublimation was performed at a temperature of − 90 °C for 1 min to decontaminate the surface of the specimen. Finally, the specimen was coated with platinum/palladium at − 140 °C for 70 s. The frozen specimen was observed in a JEOL JSM 7401F microscope operated at accelerating voltage of 1–4 kV with a working distance of around 8 mm and stage temperature at − 135 °C.

### Wax extraction

Wax was extracted from both intact leaves and isolated cuticles. In both cases, wax was extracted with chloroform from the adaxial and abaxial leaf side separately. For each 1 cm^2^ of cuticle surface, a volume of 250 μl of CHCl_3_ was applied. For intact leaves, a stainless steel syringe filter holder (Swinnex, Merck) with gaskets was used. A leaf disc (12 mm in diameter) was placed between gaskets in the holder, with the leaf side that was to be washed facing the inlet chamber. The appropriate volume of CHCl_3_ was pipetted into the inlet chamber, where it stayed in contact with the leaf surface for 30 s. After that time had elapsed, CHCl_3_ was sucked into a clean reaction vial. Each leaf disc was subjected to clean CHCl_3_ individually, and individual CHCl_3_ volumes were pooled into one sample. On average, 20 cm^2^ of leaf discs (typically from 16 to 24 leaves from 12 plants) were treated for one sample. Five samples for each variant were analysed.

Isolated cuticle discs were immersed fully in CHCl_3_ while maintaining the same ratio of CHCl_3_ volume to cuticle area. Floating cuticles, which stick well to a dry glass surface, were transferred from an aqueous solution to CHCl_3_ by using a dry glass rod (which had to be dried after each sampling) and vortexed for at least 10 s, until the cuticle was released into the CHCl_3_. The contact time of isolated cuticles with CHCl_3_ was 24 h, after which they were analysed by GC–MS. The total cuticle area and number of replicates were the same as in the experiment with washed leaves.

### Wax analysis (GC–MS)

The internal standard *n*-tetracosane (Merck, USA) was added to chloroform extracts. Samples were dried under nitrogen and derivatised in a mixture of 30 μl derivatisation agents (*N*,*O*–Bis(trimethyl)trifluoroacetamide with trimethylchlorosilane, Sigma-Aldrich, USA) and 30 μl ethylacetate (65 °C, 30 min). After cooling, 1 μl of the sample was analysed by GC–MS.

GC–MS analyses were carried out on an 8890 GC system equipped with a single quad detector (5977B, Agilent, USA). A HP-5MS UI fused silica column (Agilent, 30 m x 0.25 mm ID, 0.25 μm film thickness) was used. The flow rate of the carrier gas (Helium) was set at 1.1 ml min^−1^. The temperature program was as follows: 70 °C for 1.5 min, rising by 20 °C min^−1^ up to 160 °C, hold time 0 min, rising by 15 °C min^−1^ up to 280 °C, hold time 25 min. The injector and transfer line temperatures were 270 °C and 280 °C, respectively. The MS detector was operated in EI mode, scanning in the range of 15–650 amu. The compounds identified were C16–C30 acids, C24–C30 alcohols and C20–C33 alkanes.

### Statistical analysis

The t-test was used to determine if group means could be considered to be statistically different. The mean, standard deviation and t-test values were computed using software Origin 2018b (OriginLab Corporation, USA).

## Data Availability

The datasets generated and/or analysed in the current study are available from the corresponding author on reasonable request.
